# ERK signaling mediates CaSR-promoted axon growth

**DOI:** 10.1016/j.neulet.2015.07.019

**Published:** 2015-08-31

**Authors:** Thomas N. Vizard, Michael Newton, Laura Howard, Sean Wyatt, Alun M. Davies

**Affiliations:** School of Biosciences, Biomedical Building, Museum Avenue, Cardiff CF10 3US, UK

**Keywords:** Extracellular calcium-sensing receptor, Axon growth, Development, Sympathetic neuron, Extracellular-regulated kinase

## Abstract

The extracellular calcium-sensing receptor (CaSR) is a G-protein coupled receptor that monitors the systemic extracellular free ionized calcium level ([Ca^2+^]_o_) in organs involved in systemic [Ca^2+^]_o_ homeostasis. CaSR is widely expressed in the nervous system and its activation promotes axon and dendrite growth during development, but the mechanism by which it does this is not known. Here we show that enhanced axon growth and branching from cultured embryonic sympathetic neurons by activation of the endogenous CaSR depends on the presence of nerve growth factor (NGF). Our observation that activation of overexpressed CaSR promotes axon growth in NGF-free medium has enabled us to investigate CaSR downstream signaling contributing to axon growth in the absence of NGF signaling. We show that activation of overexpressed CaSR leads to activation of ERK1 and ERK2, and pharmacological inhibition of CaSR-dependent ERK1/ERK2 activation prevents CaSR-dependent axon growth. Analysis of axon growth from cultured neurons expressing deletion mutants of the CaSR cytoplasmic tail revealed that the region between alanine 877 and glycine 907 is required for promoting axon growth that is distinct from the high-affinity filamin-A binding site that has previously been implicated in ERK1/ERK2 activation.

## Highlights

• We have investigated how CaSR activation enhances sympathetic axon growth.
• CaSR activation promotes phosphorylation of ERK1 and ERK2.
• Inhibition of ERK1/ERK2 phosphorylation blocks CaSR-promoted axon growth.
• CaSR-promoted axon growth requires a discrete region of the cytoplasmic domain.

## 1 Introduction

The CaSR plays a crucial role in monitoring and maintaining [Ca^2+^]_o_ within very narrow physiological limits and is conspicuously expressed in the tissues and organs involved in systemic calcium homeostasis [1]. The CaSR is also widely expressed in the peripheral and central nervous system, where it has been implicated in a diversity of functions [2]. These include regulating axon and dendrite growth [3], the migration and/or maintenance of hypothalamic GnRH neurons [4] and the regulation of neuronal excitability and synaptic transmission [5,6].

The molecular mechanisms by which CaSR exerts its effects on neurons are poorly understood. The CaSR is a member of the C family of G-protein coupled receptors that associates with three main heterotrimeric G protein complexes, G_q/11_, G_i/o_ and G_12/13,_ and thereby modulates the activity of a wide variety of downstream signaling networks, including PLC-mediated Ca^2+^ mobilization, cAMP, Rho kinase and the MAP kinases ERK1/2, p38 and JNK [7]. The aim of this study was to ascertain how CaSR activation influences axon growth and branching using the well-characterized, experimentally tractable sympathetic neurons of the mouse superior cervical ganglion (SCG) [8]. Previous work has shown that expression of the CaSR peaks in these neurons in the immediate perinatal period and that activating the CaSR during this stage of development enhances NGF-promoted axon growth, and that this is important for the establishment of the appropriate level of sympathetic innervation *in vivo* [3]. Our demonstration that activation of overexpressed CaSR enhances axon growth in the absence of NGF has enabled us to investigate how the CaSR influences axon growth without the complication of concomitant NGF signaling. We show that CaSR-promoted ERK activation contributes to CaSR-promoted axon growth and identify the region of the CaSR C-terminal domain required for axon growth.

## 2 Materials and methods

### 2.1 Neuron cultures

Dissociated cultures of SCG neurons from CD-1 mice were grown on poly-ornithine/laminin coated 35 mm tissue culture dishes (Greiner) in Hams F14 medium [9] with 0.25% Albumax I (Invitrogen). Survival was estimated as described [9]. The neurite arbors of non-transfected neurons were labelled with calcein-AM (1:1000, Invitrogen). Neurons transfected with plasmids encoding full-length CaSR or CaSR mutants were co-transfected with a YFP plasmid. Fast-Sholl analysis was carried out on imaged neurons [10].

### 2.2 Plasmids

The pFLCaSR plasmid was generated by cloning the open reading frame of human CaSR into pcDNA3.1. The pG907stopCaSR and pA877stopCaSR plasmids were generated by site directed mutagenesis. Transfection was carried out using the Neon Transfection system (Invitrogen).

### 2.3 Immunocytochemistry

Cultures were fixed in ice-cold methanol for 10 min, washed in PBS, blocked and permeabilized with 5% BSA with 0.02% Triton-X100 in PBS. The cells were incubated with primary antibody in 1% BSA at 4 °C for 18 h. The primary antibodies were: anti-βIII tubulin (Promega, 1:1000), anti-CaSR to the CaSR N-terminal sequence (Imgenex, 1:1000), anti-phospho ERK1/2 and anti-total ERK1/2 (Cell Signaling Technology, 1:100). After washing, the cells were incubated with appropriate secondary antibodies conjugated to either Alexa-488 or Alexa-546 (Invitrogen), 1:600 for 90 min. Staining intensity was quantified using pixel intensity using the Volocity software (PerkinElmer).

## 3 Results

### 3.1 CaSR-promoted neurite growth is NGF-dependent

Previous work has shown that activating the CaSR in cultured SCG neurons with elevated levels of [Ca^2+^]_o_ enhances NGF-promoted axon growth in the immediate perinatal period [3]. To ascertain whether or not CaSR activation is able to enhance neurite growth independently of NGF, we compared neurite growth from E18 SCG neurons cultured with and without NGF in media containing 2.3 mM (maximally-activating) and 0.7 mM (minimally-activating) levels of [Ca^2+^]_o_ [3]. Because E18 SCG neurons are dependent on NGF for survival, we added a broad-spectrum caspase inhibitor (Boc-D-FMK) to the medium to prevent apoptosis.

**Fig. 1:**
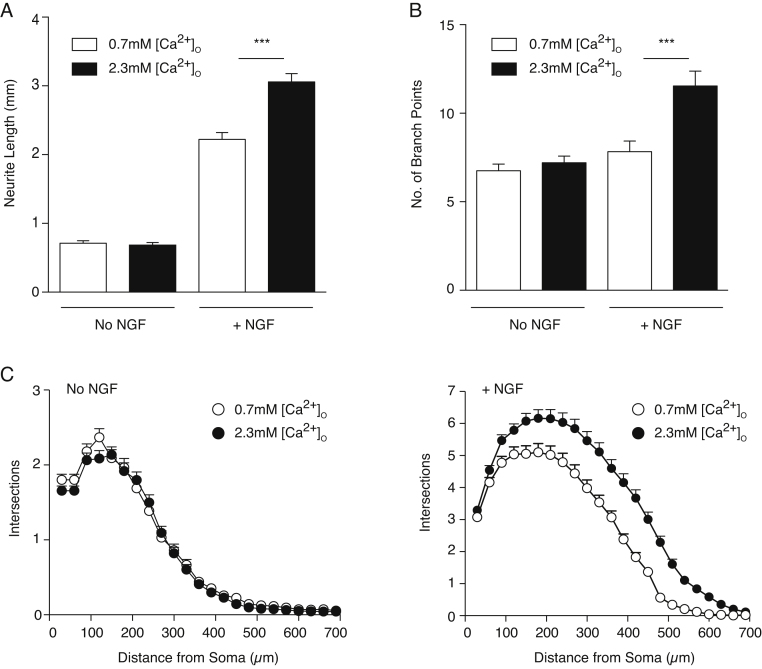
CaSR-promoted neurite growth is NGF-dependent. Neurite length (A), branch point number (B) and Sholl profiles (C) of E18 SCG neurite arbors after 24 h with and without 10 ng/ml NGF in media containing either 0.7 mM or 2.3 mM [Ca^2+^]_o_. All cultures received 50 μM Boc-D-FMK. Mean ± sem of data from 231 to 263 neurons per condition. ***P < 0.001, two-tailed, unpaired *t*-test.

In accordance with published observations [3], the neurite arbors of NGF-supplemented neurons grown in medium containing 2.3 mM [Ca^2+^]_o_ were much larger and more branched than those of neurons grown with 0.7 mM [Ca^2+^]_o_. There were highly significant differences in neurite length (Fig. 1A) and branch point number (Fig. 1B), and the Sholl profiles displayed clear differences in NGF-supplemented cultures (Fig. 1C). In contrast, the size and complexity of the neurite arbors of neurons grown without NGF were not significantly different in media containing 0.7 mM and 2.3 mM [Ca^2+^]_o_ (Figs. 1A–C). These findings suggest that activation of the endogenous CaSR is insufficient to enhance the low level of neurite growth that occurs in the absence of NGF, but enhances the magnitude of NGF-promoted neurite growth.

### 3.2 Over-expression of CaSR promotes neurite growth in the absence of NGF

**Fig. 2:**
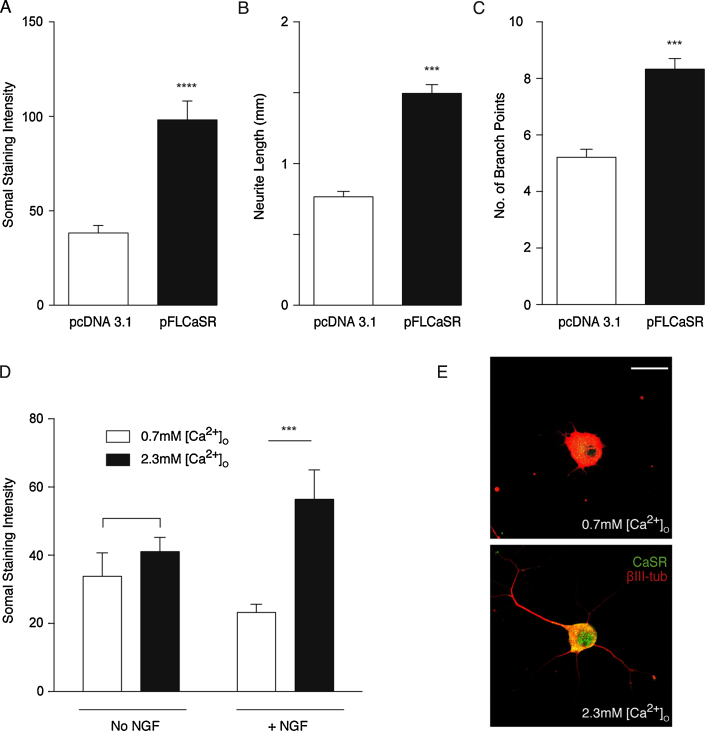
Over-expression of CaSR promotes neurite growth in the absence of NGF. (A) Quantification of CaSR immunofluorescence in E18 SCG neurons 24 h after transfection with either pFLCaSR or pcDNA3.1, mean ± sem (****P < 0.0001, unpaired *t*-test with Welch’s correction, *n* = 24 cells per condition). Total length (B) and branch point number (C) of the neurite arbors of E18 SCG neurons transfected with either pcDNA3.1 or pFLCaSR and cultured without NGF for 24 h in medium containing 2.3 mM [Ca^2+^]_o_. All cultures received 50 μM Boc-D-FMK. Mean ± sem of data from 592 to 620 neurons per condition. ***P < 0.001, two-tailed, unpaired *t*-test. (D) Quantification of CaSR immunofluorescence in non-transfected SCG neurons cultured for 24 h with and without 10 ng/ml NGF in media containing either 0.7 mM or 2.3 mM [Ca^2+^]_o_. ***P < 0.001, ANOVA with Bonferroni’s post-hoc test (*n* = 24 per condition). (E) Representative images of SCG neurons double labelled for CaSR and βIII-tubulin after 24 h in media containing 10 ng/ml NGF with either 0.7 mM or 2.3 mM [Ca^2+^]_o_. Scale bar = 20 μm.

The requirement for NGF in CaSR-promoted neurite growth complicates investigation of the signaling pathways downstream of CaSR that mediate this effect. Because enhancement of neurite growth by CaSR activation is only observed in SCG neurons at the developmental peak of CaSR expression [3], we tested whether overexpression of CaSR would enhance neurite growth in the absence of NGF. Robust high-level CaSR expression was achieved by transfecting neurons with a pcDNA3.1 vector containing full-length CaSR (pFLCaSR). Quantification of CaSR immunofluorescence confirmed increased CaSR expression in neurons transfected with pFLCaSR (Fig. 2A).

In the absence of NGF, the neurite arbors of E18 SCG neurons transfected with pFLCaSR were significantly larger than those of control transfected neurons in medium containing 2.3 mM [Ca^2+^]_o_ (Fig. 2B, and C). There was no significant difference in neurite arbor size between pFLCaSR transfected and control transfected neurons in medium containing 0.7 mM [Ca^2+^]_o_. All cultures were supplemented with Boc-D-FMK to prevent neuronal death in the absence of NGF. These findings suggest that overexpression of CaSR enhances neurite growth in NGF-free medium containing activating levels of [Ca^2+^]_o_.

Given the above results, we explored the possibility that up-regulation of CaSR expression contributes to enhanced neurite growth from non-transfected neurons grown with NGF and activating levels of [Ca^2+^]_o_. We cultured E18 SCG neurons with and without NGF in media containing either 0.7 or 2.3 mM [Ca^2+^]_o_ and estimated the relative levels of CaSR immunofluorescence after 24 h. All cultures received Boc-D-FMK to prevent apoptosis. There was no significant difference in CaSR immunofluorescence in neurons cultured with either 0.7 or 2.3 mM [Ca^2+^]_o_ in the absence of NGF (Fig. 2D). However, in the presence of NGF, the level of CaSR immunofluorescence was over two-fold higher in neurons cultured in medium containing 2.3 mM [Ca^2+^]_o_ than in neurons cultured in medium containing 0.7 mM [Ca^2+^]_o_ (Fig. 2D). Images of CaSR-labelled neurons cultured with NGF in medium containing either 0.7 mM or 2.3 mM [Ca^2+^]_o_ are illustrated in Fig. 2E. This suggests that CaSR expression is upregulated in medium containing NGF and activating levels of [Ca^2+^]_o_ and that this in turn contributes to the enhanced neurite growth observed from late fetal neurons cultured under these conditions.

### 3.3 ERK1/ERK2 activation by CaSR over-expression contributes to neurite growth

To elucidate the molecular mechanism underlying the enhancement of neurite growth from SCG neurons by activated CaSR, we explored a common link in intracellular signaling between CaSR signaling and neurite growth. ERK1 and ERK2 are activated by the CaSR in parathyroid cells, fibroblasts and kidney cell lines [11–15] and NGF-promoted ERK1/ERK2 activation in PC12 cells and SCG neurons contributes to the neurite growth response [16–20].

**Fig. 3:**
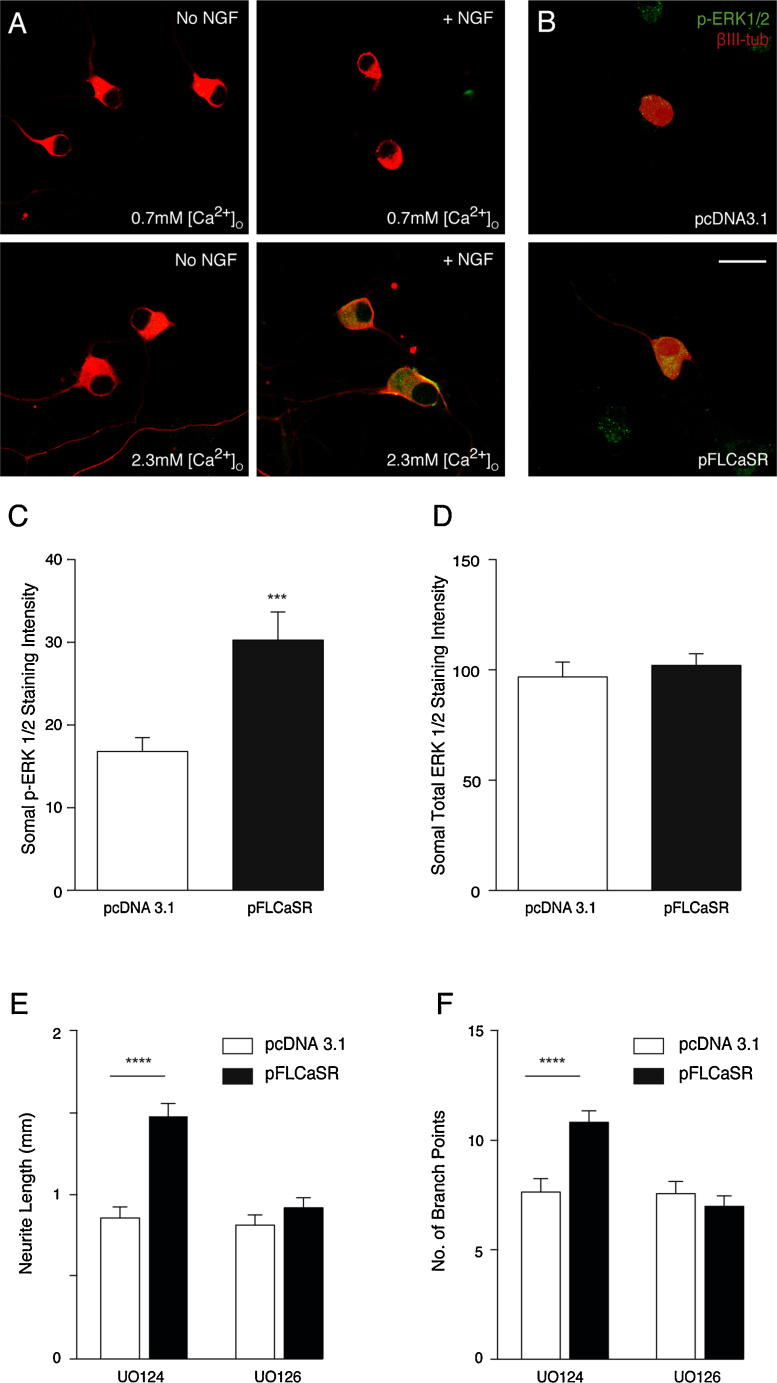
ERK1/ERK2 activation by CaSR contributes to neurite growth. (A) Images of non-transfected E18 SCG neurons double labelled for phospho-ERK1/ ERK2 and βIII-tubulin after 24 h with and without 10 ng/ml NGF in media containing either 0.7 mM or 2.3 mM [Ca^2+^]_o_. (B) Images of double labelled E18 SCG neurons after 24 h incubation in NGF-free medium containing 2.3 mM [Ca^2+^]_o_ after transfection with either pcDNA3.1 or pFLCaSR. Scale bar = 20 μm. (C and D) Quantification of phospho-ERK1/ERK2 immunofluorescence (C) and total ERK1/ERK2 immunofluorescence (D) in E18 SCG neurons cultured for 24 h in NGF-free medium containing 2.3 mM [Ca^2+^]_o_ plus 50 μM Boc-D-FMK transfected with either pFLCaSR or an pcDNA3.1. Mean ± sem of data from 40 neurons per condition. ***P < 0.001, unpaired *t*-test with Welch’s correction. Total length (E) and branch point number (F) of E18 SCG neurons cultured without NGF for 24 h in media containing 2.3 mM [Ca^2+^]_o_ plus 50 μM Boc-D-FMK and either 10 μM U0124 or 10 μM U0126 after transfection with either pFLCaSR or pcDNA3.1. Mean ± sem of data from 214 to 249 neurons per condition. ****P < 0.0001, ANOVA with Bonferroni’s post-hoc test.

To investigate whether ERK1/ERK2 signaling contributes to CaSR-promoted neurite growth from E18 SCG neurons, we used immunofluorescence to estimate the relative levels of phospho-ERK1/ERK2 in neurons. Phospho-ERK1/ERK2 immunofluorescence was clearly elevated in NGF-supplemented neurons grown in 2.3 mM [Ca^2+^]_o_ medium compared with NGF-supplemented neurons grown in 0.7 mM [Ca^2+^]_o_ medium and neurons grown in NGF-free medium containing either 0.7 mM or 2.3 mM [Ca^2+^]_o_ (Fig. 3A). There were no differences in total ERK1/ERK2 immunofluorescence in neurons grown under these different conditions (not shown). This suggests that ERK1/ERK2 signaling is elevated in neurons grown with NGF and activating levels of [Ca^2+^]_o_.

We subsequently examined whether CaSR overexpression can activate ERK1/ERK2 in E18 SCG neurons grown in NGF-free medium. The level of phospho-ERK1/ ERK2 immunofluorescence was clearly elevated in neurons overexpressing CaSR compared with control transfected neurons (Fig. 3B). Phospho-ERK1/ERK2 immunofluorescence in neurons transfected with pFLCaSR after 24 h in medium containing 2.3 mM [Ca^2+^]_o_ without NGF revealed a significant elevation compared with control-transfected neurons (Fig. 3C), whereas immunofluorescence for total ERK1/ERK1 was not significantly different (Fig. 3D).

To test whether ERK1/ERK2 activation contributes to the enhanced neurite growth brought about by CaSR over-expression, we examined whether U0126, a selective MEK1/MEK2 inhibitor that interferes with activation of ERK1/ERK2 by MEK1/MEK2 [21], could prevent the increase in CaSR-promoted neurite growth. In these experiments, we plated pFLCaSR-transfected and control-transfected SCG neurons in NGF-free medium containing Boc-D-FMK and either U0126 or its inactive analog U0124. Quantifying neurite arbor size and complexity 24 h later revealed that U0126, but not U0124, completely prevented enhanced neurite growth accompanying CaSR overexpression (Fig. 3E and F). Neither U0126 nor U0124 affected neuronal survival in these cultures (not shown). This suggests that MEK/ERK signaling contributes to CaSR-promoted neurite growth.

### 3.4 A discrete region of the CaSR cytoplasmic tail is required for enhanced neurite growth

Because many of the signaling functions of the CaSR are dependent on the C-terminal cytoplasmic domain [22,23], we transfected E18 SCG neurons with plasmids that express C-terminal truncation mutants and quantified neurite growth after 24 h culture in NGF-free medium containing 2.3 mM [Ca^2+^]_o_. The G907stop CaSR mutant lacks the high-affinity binding site for filamin-A [15] and a PKC phosphorylation site. The A877stop CaSR mutant lacks additional PKC phosphorylation sites, a PKA phosphorylation site, arginine rich motifs and a low-affinity binding site for filamin-A [15,23].

**Fig. 4:**
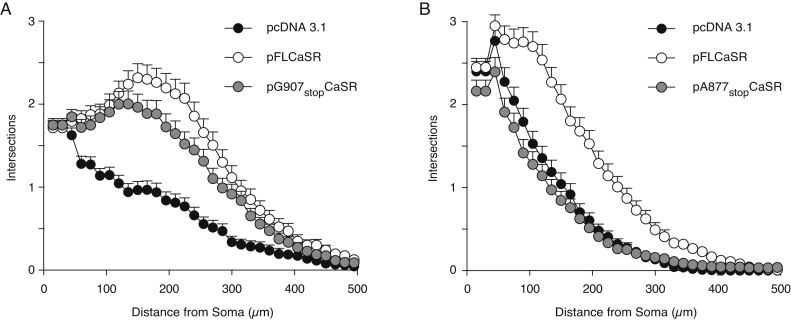
A discrete region of the CaSR cytoplasmic tail is required for enhanced neurite growth. (A) Sholl plots of E18 SCG neurite arbors after 24 h in NGF-free medium containing 2.3 mM [Ca^2+^]_o_ plus 50 μM Boc-D-FMK after transfection with either pcDNA3.1, pFLCaSR or pG907stopCaSR (data from 223 to 259 neurons per condition). (B**)** Neurons transfected with either pcDNA3.1, pFLCaSR or pA877stopCaSR (data from 182 to 210 neurons per condition).

Sholl analysis showed that the neurite arbors of E18 SCG neurons transfected with the pG907stopCaSR expression plasmid were of similar size to those of neurons transfected with pFLCaSR, whereas the neurite arbors of control-transfected neurons were very much smaller (Fig. 4A). There were no significant differences in neurite length and branch point number between pG907stopCaSR-transfected and pFLCaSR-transfected neurons (*P* = 0.9, ANOVA, data not shown), whereas control-transfected neurons were significantly shorter and less branched (*P* < 0.0001, Kruskal–Wallis test, data not shown). In contrast, the neurite arbors of pA877stopCaSR-transfected neurons were no larger than those of control-transfected neurons (Fig. 4B), and there were no significant differences in neurite length and branch point number between these groups (*P* = 0.8, ANOVA). This suggests that a region between residues alanine 877 and glycine 907 is required for CaSR-enhanced neurite growth.

## 4 Discussion

Previous work has shown that the CaSR regulates neurite growth and tissue innervation in the developing sympathetic nervous system [3]. In the current study, we have investigated how CaSR activation enhances neurite growth from SCG neurons. Our observation that activating levels of [Ca^2+^]_o_ enhance axon growth and branching from cultured SCG neurons overexpressing the CaSR in the absence of NGF has enabled us to investigate how CaSR signaling affects neurite growth without the confounding effect of NGF signaling.

We focused on a common link in intracellular signaling between CaSR signaling and the regulation of neurite growth. Several studies have shown that CaSR activation in a variety of cell types, including parathyroid cells, fibroblasts and kidney cell lines, leads to phosphorylation and activation of ERK1 and ERK2 [11–15] and ERK1/ERK2 activation in neurons and neural cell lines by NGF contributes to the neurite growth response [16–20].

Two observations suggest that ERK1/ERK2 activation is a key step by which CaSR activation enhances neurite growth. First, activating but not minimally-activating levels of [Ca^2+^]_o_ promote phosphorylation of ERK1 and ERK2 in SCG neurons overexpressing the CaSR. Second, pharmacological inhibition of the kinases that lie upstream of ERK1 and ERK2, MEK1 and MEK2, prevents activating levels of [Ca^2+^]_o_ enhancing axon growth. While these observations do not rule out the possibility that other signaling pathways activated by the CaSR may contribute to its influence on neurite growth, these findings suggest that ERK1/ERK2 activation is a necessary step in the neurite growth-promoting effects of the CaSR.

To determine the region of the CaSR required for enhancing neurite growth, we overexpressed CaSR deletion mutants to ascertain the minimum length of the CaSR intracellular domain required for enhancing neurite growth in the presence of activating levels of [Ca^2+^]_o_. Because activation of ERK1/ERK2 by the CaSR depends on direct interaction of the CaSR carboxyl terminus with the cytoskeletal scaffold protein filamin A [13,15,24], we used deletion mutants that lack one or both binding sites for filamin A. Overexpression of the G907stop mutant, which lacks the high-affinity filamin-A binding site [15], was just as effective as overexpressed full-length CaSR in promoting neurite growth in medium containing activating levels of [Ca^2+^]_o_, indicating that the high-affinity filamin A binding site is not required for CaSR-promoted neurite growth. It is possible that this partially truncated mutant may enhance neurite growth by a mechanism that differs from that of full-length CaSR. A recent study in HEK-293 cells has also shown that the high-affinity filamin A binding site is also dispensable for ERK1/ERK2 activation by the CaSR [15]. Overexpression of the A877stop CaSR mutant failed to enhance neurite growth with activating levels of [Ca^2+^]_o_, suggesting that the region between residues alanine 877 and glycine 907 is required for CaSR-enhanced neurite growth. This region not only possesses a low-affinity filamin A binding site [15], but also contains PKC phosphorylation sites [25–28] and residues necessary for PI-PLC activation [29]. These findings are consistent with the possibility that the low-affinity filamin A binding site is important, but raise the possibility that other sites within this short stretch of the C-terminal domain contribute to CaSR-promoted neurite growth. Further molecular dissection of this region will be required to address this possibility.
